# Giant dissecting aneurysm of the superior mesenteric artery with distal branch involvement and true lumen collapse: a case report

**DOI:** 10.3389/fmed.2026.1836483

**Published:** 2026-06-09

**Authors:** Chuwen Chen, Yiyuan Li, Bin Huang, Xiyang Chen

**Affiliations:** Division of Vascular Surgery, Department of General Surgery, West China Hospital, Sichuan University, Chengdu, Sichuan, China

**Keywords:** dissecting aneurysm, impending rupture, open surgical repair, prosthetic graft reconstruction, superior mesenteric artery dissection, true lumen collapse

## Abstract

**Background:**

Isolated superior mesenteric artery dissecting aneurysm (ISMADA) is rare and may lead to fatal outcomes. This case report presents the surgical management of a patient with a giant ISMADA complicated by suspected impending rupture.

**Case presentation:**

A 70-year-old man was admitted with a 2-year history of intermittent abdominal pain that had worsened over the preceding 2 days. Examination revealed a 5 × 5 cm pulsatile periumbilical mass with tenderness and mild lower abdominal tenderness with questionable rebound. Computed tomography angiography showed a 4.8 cm isolated superior mesenteric artery dissecting aneurysm involving distal branches, with severe true lumen compression. Laboratory tests showed mild leukocytosis. Emergency resection of the aneurysm with prosthetic graft reconstruction of the superior mesenteric artery was performed in a hybrid operating room. Postoperative angiography confirmed graft patency. The patient resumed oral intake on postoperative day 4 and was discharged uneventfully on day 8. At 30-month follow-up, he remained asymptomatic without abdominal or gastrointestinal complaints.

**Conclusion:**

Although rare, isolated superior mesenteric artery dissection can progress to aneurysmal degeneration, rupture, and hemorrhage. Most uncomplicated ISMAD cases can be managed conservatively, and endovascular therapy is feasible in anatomically suitable patients; however, open surgical repair remains indispensable in selected complex cases.

## Introduction

Isolated superior mesenteric artery dissection (ISMAD) is a visceral arterial disorder characterized by dissection confined to the superior mesenteric artery (SMA) in the absence of concomitant aortic dissection (1). ISMAD was previously considered rare, with autopsy data suggesting a prevalence of approximately 0.075% (2). However, with the widespread use of computed tomography and computed tomography angiography (CTA) in the evaluation of acute abdominal conditions, the detection rate of ISMAD has gradually increased (3). This disease shows distinct geographic and demographic features, with most reported cases originating from East Asia, particularly China, Japan, and Korea (4). ISMAD predominantly affects middle-aged and elderly men, and the dissection most commonly occurs 1 to 3 cm distal to the SMA origin (5, 6).

The etiology and pathogenesis of ISMAD remain incompletely understood. Proposed contributing factors include hypertension, smoking, structural abnormalities of the arterial wall, connective tissue disorders, fibromuscular dysplasia, and hemodynamic stress (4). In particular, the anatomic transition zone between the fixed proximal SMA and the more mobile distal segment, an increased SMA-aortic angle, and elevated local wall shear stress may play important roles in intimal injury and subsequent dissection formation (7). In addition, celiac artery compression or stenosis caused by median arcuate ligament syndrome may increase compensatory collateral flow from the SMA through the pancreaticoduodenal arcade, thereby increasing SMA flow volume and wall shear stress (8, 9). This mechanism has been proposed as a rare potential precipitating factor for ISMAD.

Clinically, ISMAD most commonly presents with sudden-onset epigastric or mid-abdominal pain, although the symptoms are nonspecific. Some patients may remain asymptomatic, whereas a small proportion may progress to intestinal ischemia, bowel necrosis, or arterial rupture. In rare cases, aneurysmal degeneration of the dissected SMA may occur; therefore, early radiologic recognition and close surveillance are essential. For asymptomatic patients or those with uncomplicated symptomatic isolated dissection of the SMA or celiac artery, conservative management is recommended as the first-line treatment, including blood pressure control, analgesia, and bowel rest. The European Society for Vascular Surgery and Society for Vascular Surgery guidelines (10, 11) do not recommend routine upfront endovascular or open vascular reconstruction in these patients. However, endovascular revascularization is recommended for patients who fail medical therapy or are suspected of having intestinal ischemia. Follow-up imaging after conservative treatment or stent placement is also advised to monitor for aneurysm formation or progressive stenosis (10, 11).

Nevertheless, the management of complex SMA dissecting aneurysms remains challenging. Herein, we report the successful emergency treatment of a giant isolated SMA dissecting aneurysm with distal branch involvement and true lumen occlusion. This case was unusual because the aneurysm was symptomatic and associated with marked abdominal tenderness, suggesting impending rupture. In addition, the true lumen was severely compressed, multiple critical distal branches were involved, and distal perfusion was supplied by the false lumen. Therefore, endovascular exclusion alone was not feasible. A hybrid surgical approach was adopted and successfully resolved this complex vascular problem. This report was prepared in accordance with the 2023 SCARE criteria (12).

## Case presentation

**Figure 1:**
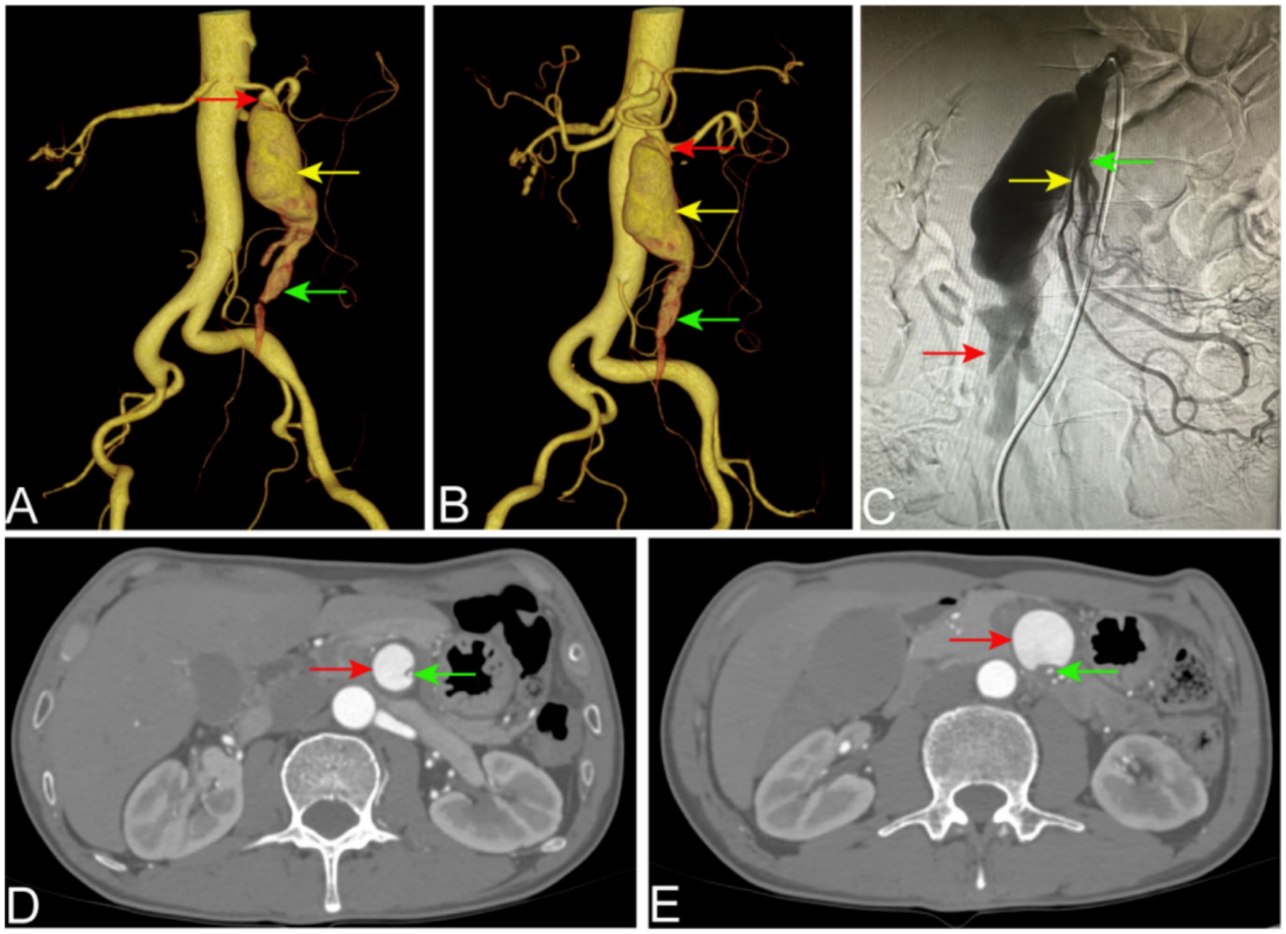
Preoperative imaging findings. **(A,B)** Emergency computed tomography angiography (CTA) demonstrated a dissecting aneurysm of the superior mesenteric artery measuring 4.2 × 4.8 cm (yellow arrow). The dissection involved the first proximal branch of the SMA (red arrow) and the distal SMA branches were supplied by the false lumen (green arrow). **(C)** Emergency angiography revealed a giant dissecting aneurysm of the superior mesenteric artery. The dissection involved the first proximal branch of the SMA (green arrow) as well as the distal SMA branches (red arrow) and the true lumen was compressed by the false lumen, appearing as a slit-like lumen (yellow arrow). **(D,E)** Axial CT images showed that the true lumen (green arrow) was nearly completely compressed and occluded by the false lumen (red arrow).

**Figure 2:**
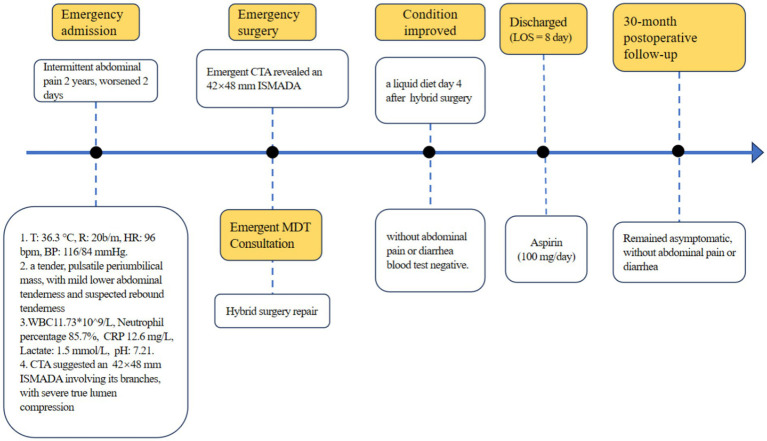
Clinical timeline of the patient’s diagnosis, treatment, and follow-up.

A 70-year-old man was admitted to the emergency department with progressively worsening abdominal pain over the preceding 2 days. He reported a 2-year history of intermittent abdominal pain, which had recently become more persistent and was accompanied by abdominal discomfort, prompting emergency presentation. His medical history was notable for hypertension of more than 2 years’ duration, treated with sustained-release nifedipine 30 mg/day. He had no history of abdominal surgery, vascular disease, connective tissue disorders, mesenteric ischemia, or aneurysmal disease. He had a 20-year smoking history of approximately 10 cigarettes per day and denied alcohol or illicit drug use. There was no family history of aneurysmal disease, vascular disorders, or hereditary connective tissue disease. On admission, his vital signs were stable, with a blood pressure of 116/84 mmHg, heart rate of 96 beats/min, respiratory rate of 20 breaths/min, and body temperature of 36.3 °C. Physical examination revealed a poorly mobile, pulsatile mass measuring approximately 5 cm × 5 cm in the periumbilical region, without a palpable thrill. The mass was tender on light palpation, and mild lower abdominal tenderness was also present, with equivocal rebound tenderness. Laboratory tests showed leukocytosis with a white blood cell count of 11.73 × 10^9^/L, a neutrophil percentage of 85.7%, and an elevated C-reactive protein level of 12.6 mg/L. The lactate level was 1.5 mmol/L, and the arterial pH was 7.21. The remaining laboratory parameters were within normal limits. Emergency CTA revealed a 4.2 × 4.8 cm ISMADA. The proximal extent of the aneurysm involved the first branch of the SMA (Figures 1A,B). The true lumen (TL) of the mid-segment of the SMA was nearly completely compressed and occluded by the false lumen (FL) (Figures 1D,E). Three to four jejunal arteries originated from the proximal portion of the true lumen (Figure 1C); however, the distal branches of the SMA were supplied by the false lumen (Figure 1C). Based on the clinical presentation and imaging findings, the patient was diagnosed with an ISMADA with suspected impending rupture, suspected bowel ischemia of uncertain chronicity, and hypertension. The patient’s clinical timeline is shown in Figure 2.

### Treatment

To optimize treatment planning, a multidisciplinary team consisting of vascular surgeons, gastrointestinal surgeons, interventional radiologists, and intensivists was convened to discuss the management strategy. First, given the patient’s persistent abdominal pain, focal tenderness over the aneurysm, and suspected rebound tenderness, the team agreed that immediate intervention was warranted, as these findings suggested possible impending rupture and ongoing intestinal ischemia. Second, the interventional radiologists considered endovascular repair to be of limited feasibility because the true lumen of the mid SMA was almost completely compressed by the false lumen, and approximately half of the major distal mesenteric branches were perfused through the false lumen. In addition, based on the elevated white blood cell count, increased neutrophil percentage, elevated C-reactive protein level, and suspected abdominal rebound tenderness, the gastrointestinal surgeons considered exploratory evaluation of the bowel necessary to assess for ischemia or necrosis. After multidisciplinary discussion, a hybrid approach was deemed safe and feasible. The planned strategy consisted of initial angiography to fully delineate the vascular anatomy and lesion characteristics, followed by immediate open repair, and finally completion angiography after open reconstruction to confirm technical success and distal perfusion.

**Figure 3:**
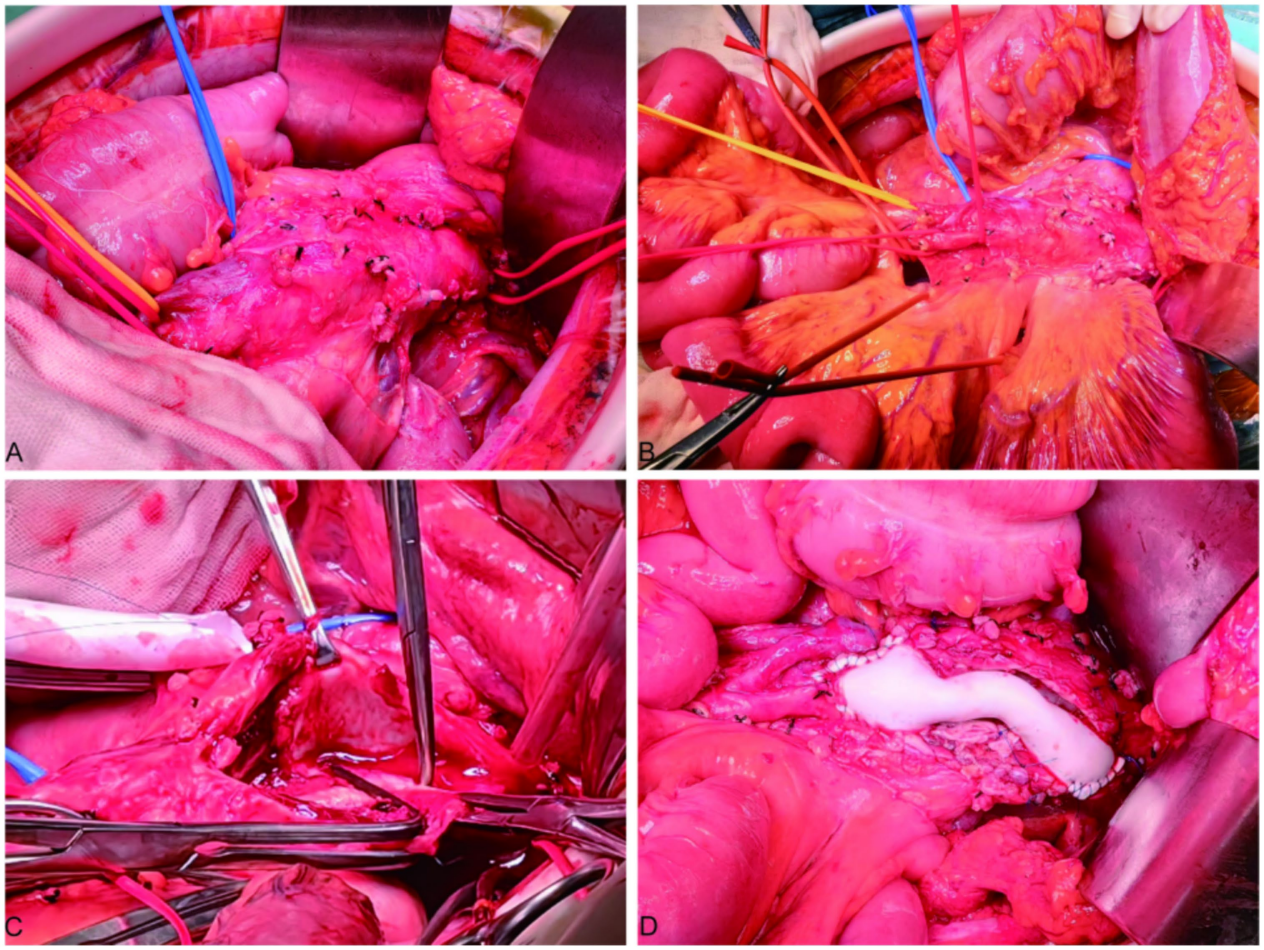
Operative imaging findings. **(A)** Exposure of the superior mesenteric artery dissecting aneurysm (green arrow) and its major branches (black arrow). **(B)** The mesentery was spread in a fan-shaped fashion, and fenestrations were created in avascular areas at approximately 7 cm intervals. Subsequently, distal mesenteric inflow was sequentially controlled using vascular clamps (green arrow). **(C)** The aneurysm sac was opened longitudinally, and the dissecting intimal flap was excised, allowing fusion of the true lumen (green arrow) and false lumen (yellow arrow). **(D)** Reconstruction of the superior mesenteric artery using a prosthetic vascular graft, with proximal anastomosis (green arrow) and distal anastomosis (black arrow).

**Figure 4:**
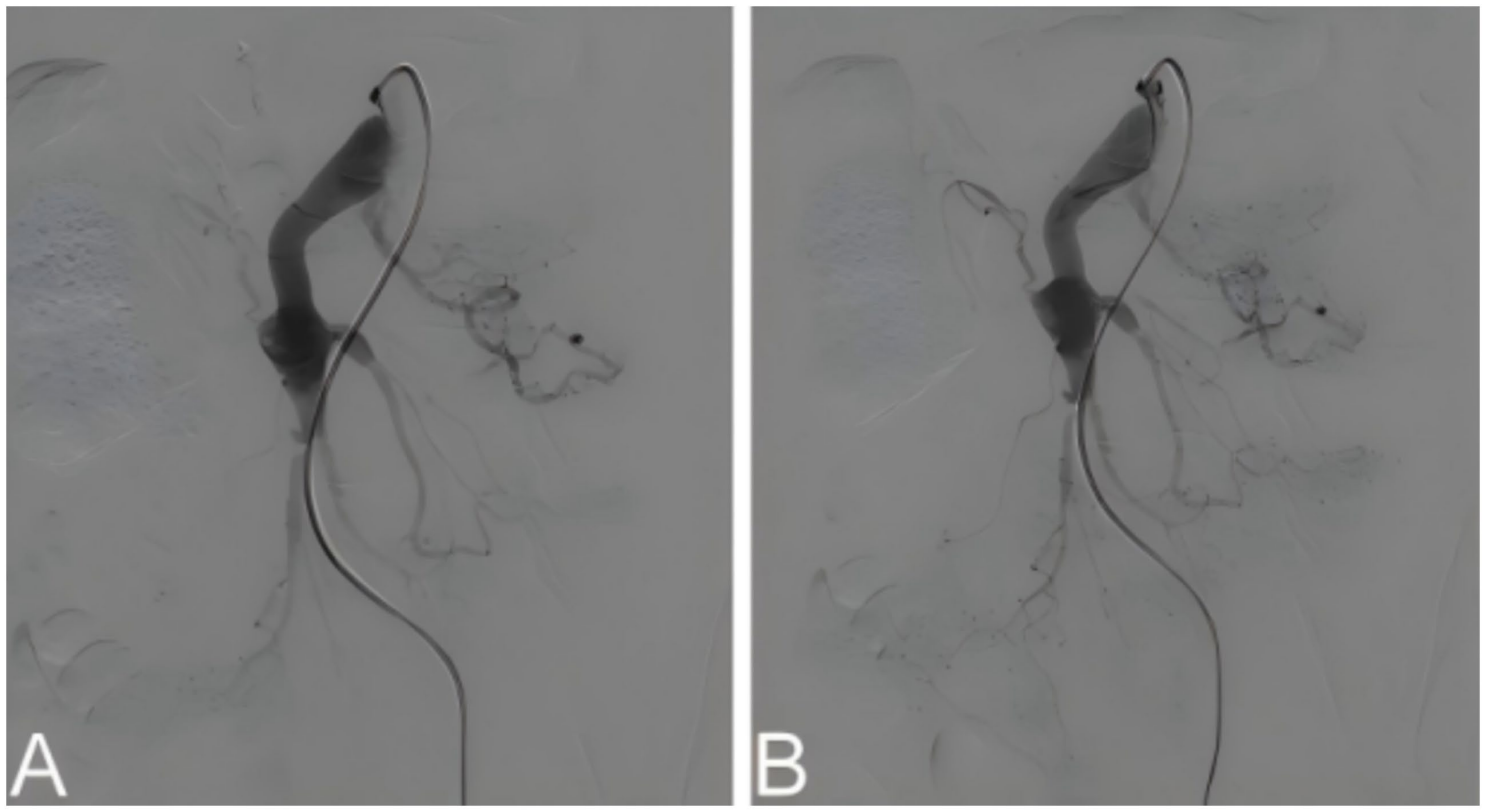
Postoperative angiography images. **(A,B)** Immediate angiography performed after resection of the superior mesenteric artery dissecting aneurysm with prosthetic graft reconstruction demonstrated successful revascularization, with good opacification of the superior mesenteric artery and its branch vessels.

Percutaneous access was obtained in the right common femoral artery, and a 5-French vascular sheath was placed. A pigtail catheter was advanced to the level of the T12 vertebral body, and diagnostic angiography was performed to delineate the SMA origin and branch anatomy. The pigtail catheter was then exchanged for an angled hydrophilic Simmons catheter, which was selectively advanced into the SMA to confirm the complex lesion architecture and branch involvement (Figure 1C). Angiography confirmed that the true lumen of the superior mesenteric artery was almost completely compressed by the false lumen, and that approximately half of the major distal mesenteric branches were perfused through the false lumen. Therefore, endovascular treatment carried substantial risks, including failure of stent expansion, loss of critical mesenteric arterial perfusion with covered stent placement, and incomplete exclusion of the dissecting aneurysm with bare-metal stent placement. Subsequently, open surgical repair was initiated. The aneurysm was exposed via a midline abdominal incision (Figure 3A), and the bowel showed no evidence of ischemia. The proximal SMA was accessed via a medial approach to the duodenum and circumferentially mobilized; vessel loops were placed for proximal control. This dissection included jejunal branches arising from the TL as well as branches supplied by the FL. Because the dissecting aneurysm was densely adherent to the small-bowel mesentery, and to minimize the risk of tissue injury, the aneurysm sac was not circumferentially dissected. Distal vascular control was achieved without individual dissection of each branch. Instead, the mesentery was spread in a fan-shaped fashion and fenestrations were created in avascular areas of the mesentery at approximately 7-cm intervals, through which vessel loops were placed for distal control (Figure 3B). After all preparations were completed, proximal SMA clamping was performed first, followed by sequential occlusion of distal mesenteric inflow to prevent retrograde perfusion. The dissecting aneurysm sac was then opened, and the proximal intimal tear was resected to unify the TL and FL (Figure 3C). Proximal reconstruction was performed using a 6-mm expanded polytetrafluoroethylene (ePTFE) graft in an end-to-side anastomosis to restore perfusion to the jejunal arteries. Distally, an end-to-side anastomosis was similarly performed to revascularize branches originating from the FL (Figure 3D). The total SMA ischemic time was 20 min. Upon completion of reconstruction, strong pulsations were observed in the proximal jejunal arteries, distal SMA branches, and the ileocolic artery. The bowel appeared viable, with normal coloration and turgor (Figure 3D). Completion angiography was performed via the right femoral sheath using a hydrophilic Simmons catheter selectively advanced into the SMA, confirming excellent opacification of the proximal jejunal arteries, distal SMA branches, and the ileocolic artery (Figures 4A,B).

### Patient perspective and follow-up

The patient resumed a liquid diet on postoperative day 4 without abdominal pain or diarrhea, and fecal occult blood testing was negative. The postoperative course was uneventful, and he was discharged on postoperative day 8. During hospitalization, the patient tolerated recovery well and reported no recurrent abdominal symptoms before discharge. During 30 months of telephone follow-up, he remained asymptomatic, without abdominal pain, diarrhea, gastrointestinal discomfort, or other related symptoms. However, follow-up imaging could not be obtained because of geographic constraints despite repeated attempts to arrange further assessment. Written informed consent was obtained from the patient and his family.

## Discussion

Most patients with uncomplicated ISMAD have favorable short- and mid-term outcomes with conservative management, particularly those who are asymptomatic or mildly symptomatic. The current therapeutic goals for ISMAD are mainly to relieve symptoms and prevent serious complications, including bowel ischemia, bowel necrosis, aneurysmal degeneration, and rupture (13). Both the Society for Vascular Surgery and European Society for Vascular Surgery guidelines emphasize that close observation with serial imaging follow-up may be considered for SMA dissection or dissecting aneurysm in the absence of intractable pain, bowel ischemia, signs of rupture, or other features suggestive of disease progression (10, 11).

During follow-up, most ISMAD lesions may undergo varying degrees of luminal remodeling (14). Based on imaging findings, remodeling is generally classified as complete or incomplete (15). Complete remodeling refers to restoration of the arterial morphology to a near-normal configuration, usually accompanied by false lumen thrombosis or obliteration, true lumen expansion, and relief of stenosis (16). Incomplete remodeling is characterized by persistent false lumen patency, residual stenosis, partial thrombosis, ulcer-like projection, aneurysmal dilatation, or progressive enlargement of the lesion during follow-up (14, 15, 17). Luminal remodeling is not only an imaging outcome but may also reflect a dynamic balance among false lumen pressure, intimal flap stability, branch perfusion, distal outflow, and local hemodynamic conditions (14, 16). Therefore, favorable remodeling may support continued surveillance, whereas persistent false lumen perfusion, progressive aneurysmal enlargement, severe true lumen compression, branch vessel involvement, or recurrent symptoms may indicate lesion instability and should prompt consideration of further intervention (10, 11). Uncontrolled hypertension, smoking, and treatment modality may all influence vascular remodeling (18, 19). It should be noted that, unlike degenerative true aneurysms, the rupture risk of dissecting aneurysms may not be determined solely by maximum diameter. Persistent pressurization of the false lumen, localized intimal tears, weakening of the medial layer, increased eccentric wall stress, and branch-related alterations in outflow may all contribute to disease progression. Therefore, even small dissecting aneurysms may carry a high risk if they are associated with persistent false lumen perfusion, rapid morphologic change, perivascular inflammatory changes, localized tenderness, or clinical symptoms suggestive of impending rupture. Current guidelines provide no clear recommendations for small-diameter ISMAD-related dissecting aneurysms with high-risk features (10, 11). Thus, in clinical practice, risk stratification should not rely on aneurysm diameter alone but should incorporate symptoms, physical findings, CTA morphologic changes, false lumen status, the degree of true lumen compression, and branch perfusion. In addition, previously reported rates of complete and incomplete remodeling vary substantially; therefore, regular imaging surveillance remains essential regardless of whether patients are treated conservatively, endovascularly, or surgically.

Although aneurysmal degeneration in ISMAD is uncommon, rupture can have devastating consequences. Previous studies of visceral artery aneurysms have reported rupture rates of 25–40%, with postrupture mortality approaching 76% (20, 21). With the widespread use of CTA, ISMADA is being recognized more frequently. However, the optimal treatment strategy for complex symptomatic lesions remains challenging (3). In recent years, endovascular treatment has been widely used in anatomically suitable patients because of its minimally invasive nature, low perioperative morbidity, and shorter hospital stay (13, 15). Nevertheless, its feasibility is highly dependent on lesion anatomy. Long-segment dissection, severe true lumen compression or occlusion, distal branch involvement, distal perfusion dependent on the false lumen, and inadequate proximal or distal landing zones may compromise both effective aneurysm exclusion and preservation of mesenteric perfusion. In such circumstances, open surgical reconstruction remains a reliable and flexible treatment option. In the present case, the patient had persistent abdominal pain, localized tenderness, and suspected signs of peritoneal irritation, suggesting a high-risk condition with possible impending rupture or concomitant bowel ischemia. Emergency intervention was therefore required. Because the true lumen was severely compressed by the false lumen and a substantial proportion of important distal mesenteric branches were supplied by the false lumen, endovascular treatment alone was considered unsuitable. A bare-metal stent would have been unlikely to completely exclude the dissecting aneurysm, whereas a covered stent could have occluded important mesenteric branches and caused bowel ischemia. In addition, severe true lumen compression would have increased the risks of inadequate stent expansion, failed aneurysm exclusion, and loss of critical distal perfusion. Open repair was also technically challenging. First, severe compression of the true lumen by the false lumen made accurate intraoperative identification of the true lumen difficult. An incorrect distal anastomotic site could have resulted in catastrophic bowel ischemia. To reduce this risk, we opened the aneurysm sac and resected the intimal flap, thereby converting the true and false lumens into a common outflow channel and facilitating reliable reconstruction. Second, adequate exposure and reconstruction required temporary interruption of mesenteric blood flow. Individual dissection and control of distal branches would have been time-consuming and could have increased the risk of branch injury. Therefore, we used a fan-shaped mesenteric exposure technique, creating multiple windows in avascular areas to obtain effective distal vascular control. Given the limited ischemic tolerance of the bowel, rapid restoration of mesenteric perfusion was critical. Because the great saphenous vein was small in diameter and remained insufficient for reconstruction even after splicing, and because no definite evidence of intra-abdominal infection was observed intraoperatively, an expanded polytetrafluoroethylene graft was selected for reconstruction. Under standard antiplatelet therapy, ePTFE grafts can provide acceptable patency. Proximal and distal end-to-side anastomoses allowed restoration of perfusion to the main mesenteric trunk and distal branches while minimizing ischemic time.

## Conclusion

Although giant dissecting aneurysms of the superior mesenteric artery are rare, they carry substantial risk. Careful surveillance of morphologic remodeling in ISMAD is therefore essential. Although most uncomplicated ISMAD lesions can be managed conservatively or with endovascular therapy in anatomically suitable patients, open surgical repair remains indispensable in selected complex cases. For anatomically complex SMA dissections, meticulous operative planning and individualized reconstruction strategies are key to successful management.

## Data Availability

The raw data supporting the conclusions of this article will be made available by the authors, without undue reservation.
